# Succinylation: A Functional Nexus Between Metabolic Reprogramming and Epigenetic Modifications in Cancer

**DOI:** 10.3390/molecules31050773

**Published:** 2026-02-25

**Authors:** Dan Liu, Runtian Li, Mingzhu Li, Fang Xu, Ying Liang, Yang Sun

**Affiliations:** Department of Biology, College of Basic Medicine, Heilongjiang University of Chinese Medicine, No. 24, Heping Road, Xiangfang District, Harbin 150040, China; ld20190218@163.com (D.L.); lrt_9703@163.com (R.L.); limingzhu0208@163.com (M.L.); xu-fang1234@163.com (F.X.); liangying_8889@sina.com (Y.L.)

**Keywords:** lysine succinylation, succinyl-CoA, metabolic reprogramming, epigenetic modification, cancer

## Abstract

Metabolic reprogramming and epigenetic remodeling are critical features of tumorigenesis. The process of metabolic reprogramming causes metabolites like Succinyl-CoA to accumulate. Succinylation, which depends on succinyl-CoA as the direct donor group, plays a crucial role in regulating cancer metabolism. This involves the transfer of the succinyl group to the lysine residues of substrate proteins resulting in the alteration of the conformation and function of the proteins, modulating several signaling pathways, many of them involved in metabolism. There is growing evidence that succinylation can alter the activity and stability of metabolic enzymes and reshape metabolic networks. Furthermore, it precisely regulates gene expression through the epigenetic modification mechanisms of the histones and non-histone proteins. Lysine succinylation is thus a crucial hub linking tumor metabolic reprogramming and epigenetic remodeling. This review systematically summarizes the dynamic regulatory mechanisms of lysine succinylation and its critical roles in tumor metabolic reprogramming and epigenetic regulation. In the end, we discuss the crosstalk between succinylation and other post-translational modifications (PTMs) as well as recent advances in cancer therapies targeting succinylation.

## 1. Introduction

Lysine succinylation (Ksuc) is a significant post-translational modification (PTM) characterized by the covalent attachment of the succinyl group (-CO-CH_2_-CH_2_-COOH) to the ε-amino group of lysine residues in proteins [1]. Succinylation plays a crucial role in regulating protein activity, stability, and cellular localization across various biological processes [2,3,4]. Importantly, the abundance of Ksuc is intricately linked to cellular metabolism, and its dysregulation is considered as a critical factor in tumor progression [5,6].

Metabolic reprogramming is a fundamental characteristic of tumors, and the accumulation of metabolites resulting from this process serves as a primary effector driving the malignant progression of tumors [7,8]. To meet the demands of rapid proliferation, the metabolic pathways in tumor cells are typically enhanced, leading to a significant accumulation of succinyl-CoA, an intermediate product of the tricarboxylic acid cycle (TCA cycle). Succinyl-CoA acts as a co-substrate that directly catalyzes the succinylation of various key metabolic enzymes [9,10]. This succinylation further consolidates and amplifies the abnormal state of the metabolic network by regulating the activity and stability of target enzymes, thereby creating a metabolic microenvironment that promotes tumor growth and metastasis [10,11]. Additionally, succinyl-CoA can influence epigenetic modification by modifying histones and transcription factors, which in turn regulates gene expression in an epigenetic manner through alterations in chromatin structure or modulation of protein interactions. It also participates in biological processes such as metabolic reprogramming and DNA damage repair [3,8].

In conclusion, there exists a close bidirectional relationship between tumor cell metabolism and epigenetics, with succinylation serving as the central hub that connects the two. Tumor metabolite-mediated succinylation can directly regulate the activity of metabolic enzymes while also epigenetically modulating gene expression, thereby dynamically reshaping the tumor metabolic network to establish a self-reinforcing cycle of ‘metabolism-epigenetics-metabolism.’ This review systematically summarizes the mechanistic role of succinylation in tumor metabolic and epigenetic reprogramming, aiming to provide novel theoretical perspectives for related research.

## 2. Discovery and Basic Characteristics of Succinylation

**Figure 1:**
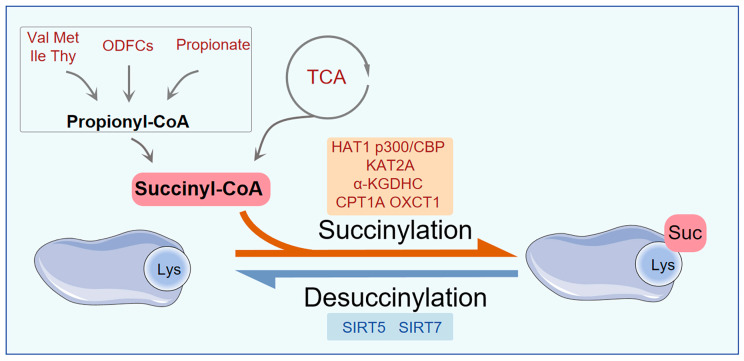
The source and regulators of succinylation. Succinyl-CoA is predominantly synthesized via the TCA cycle, although it can also originate from the transformation of propionyl-CoA. Propionyl-CoA is generated from the breakdown of valine (Val), isoleucine (Ile), methionine (Met), and thymine (Thy), as well as from the oxidation of odd-chain fatty acids (OCFAs) and propionate. HAT1, p300/CBP, KAT2A, α-KGDHC, CPT1A, and OXCT1A function as succinyltransferases that facilitate the succinylation of lysine residues. SIRT5 and SIRT7 act as desuccinylases, promoting the desuccinylation of lysine residues. Abbreviations: Succinyl-CoA, succinyl coenzyme A; HAT1, histone acetyltransferase 1; KAT2A, lysine acetyltransferase 2A; α-KGDHC, α-ketoglutarate dehydrogenase complex; CPT1A, carnitine palmitoyltransferase 1A; OXCT1, 3-oxoacid CoA-transferase 1; SIRT5, sirtuin 5; SIRT7, sirtuin 7; Suc, succinylation.

Succinylation was first reported in 2011 and has been shown to be prevalent in both prokaryotic and eukaryotic organisms. This modification involves the covalent attachment of a succinyl group (-CO-CH2-CH2-COOH) to the ε-amino group of lysine residues in proteins. This process alters the charge of lysine from +1 to −1 at physiological pH (approximately 7.4), which can disrupt existing electrostatic interactions within proteins, such as salt bridges, thereby modifying the active interface of proteins and their interactions with other molecules. Furthermore, succinylation introduces a mass shift of approximately +100.0186 Da, resulting in changes to the local spatial conformation of proteins [1]. Such alterations can affect enzyme activity and subsequently regulate various cellular signaling pathways, particularly those associated with metabolism [6,10]. The dynamic balance of protein succinylation is primarily governed by the concerted actions of succinyl donors and succinylation-regulating enzymes, which include ‘writers,’ ‘erasers,’ and ‘readers.’ This process can be mediated through both enzymatic catalysis and non-enzymatic pathways (see Figure 1) [12,13,14].

### 2.1. Non-Enzymatic Succinylation

Succinylation can occur through spontaneous non-enzymatic processes, which are regulated by the concentration of the succinyl donor, succinyl-CoA, and the pH levels [15]. In tissues with elevated levels of succinyl-CoA—such as those resulting from α-ketoglutarate (α-KG) supplementation or succinate dehydrogenase (SDH) deficiency—the levels of Ksuc correspondingly increase [16,17]. Therefore, succinyl-CoA, as a crucial metabolic intermediate, drives non-enzymatic succinylation both inside and outside mitochondria in a concentration-dependent manner.

Succinyl-CoA is primarily synthesized in the mitochondria via the TCA cycle and the conversion of propionyl-CoA [18,19]. Within the TCA cycle, α-ketoglutarate (α-KG) is directly transformed into succinyl-CoA by the α-ketoglutarate dehydrogenase complex (α-KGDHC) [20]. Additionally, a significant source of succinyl-CoA is propionyl-CoA, which is generated from the catabolism of valine (Val), isoleucine (Ile), methionine (Met), and thymine (Thy), as well as from the β-oxidation of odd-chain fatty acids (OCFAs) and propionate [19]. Propionyl-CoA is subsequently converted into succinyl-CoA through the actions of propionyl-CoA carboxylase and methylmalonyl-CoA mutase [19].

Although the classical pathway for succinyl-CoA generation is localized in the mitochondria, multiple lines of evidence indicate its presence in both the nucleus and cytoplasm, thereby supporting widespread succinylation in these compartments [21,22]. For instance, α-KGDHC can translocate to the nucleus and catalyze the production of succinyl-CoA within it [23]. Meanwhile, the succinyl-CoA ligase ADP-forming subunit β (SUCLA2) in the nucleus can also mediate succinyl-CoA generation, and a deficiency in SUCLA2 leads to decreased global protein succinylation levels [17]. In the cytoplasm, although a direct pathway for catalyzing the synthesis of succinyl-CoA is absent [24], the oxidation of long-chain fatty acids in peroxisomes can generate succinyl-CoA, which is subsequently transported to the cytoplasm as succinylcarnitine, thereby supporting the succinylation of cytoplasmic proteins [19].

### 2.2. Regulatory Enzymes of Succinylation

Lysine succinylation can occur extensively through non-enzymatic mechanisms; however, it is also subject to precise regulation through enzymatic processes. The enzymes involved in the regulation of succinylation can be categorized into three major groups: ‘writers,’ ‘erasers,’ and ‘readers.’

#### 2.2.1. Epigenetic Writers

The ‘writers’ are responsible for attaching succinyl groups to lysine residues on substrate proteins, thereby altering their spatial conformation and biological functions. Currently identified ‘writers’ include several classical histone acetyltransferases, which are primarily localized in the nucleus [25]. Histone acetyltransferase 1 (HAT1) not only regulates protein acetylation but also mediates protein succinylation independently of its acetylation activity [26]. The p300/CBP complex can catalyze the succinylation of H3K122 in HepG2 cells [27], while lysine acetyltransferase 2A (KAT2A) catalyzes the succinylation of H3K79 in pancreatic ductal adenocarcinoma (PDAC) cells [28]. Notably, nuclear α-KGDHC interacts with tyrosine 645 (Y645) of KAT2A to form a complex, enabling KAT2A to utilize succinyl-CoA, which is locally generated by α-KGDHC, for mediating H3K79 succinylation [23].

The E2k subunit of α-KGDHC in mitochondria catalyzes the succinylation of mitochondrial proteins in an α-ketoglutarate-dependent manner [29,30]. Inhibition of α-KGDHC significantly reduces the succinylation levels of these proteins [29]. Carnitine palmitoyltransferase 1A (CPT1A) exhibits succinyltransferase activity both in vivo and in vitro [31]. Recent studies have demonstrated that 3-oxoacid CoA-transferase 1 (OXCT1), which is involved in ketone body catabolism, also possesses succinyltransferase activity. Notably, OXCT1 can catalyze the succinylation of the anticancer protease serine beta-lactamase-like protein(LACTB) at K284, consequently impairing its protease function [5].

#### 2.2.2. Epigenetic Erasers

The ‘erasers’ function to remove existing succinyl groups from substrates. In eukaryotes, the Sirtuin family consists of a class of nicotinamide adenine dinucleotide (NAD^+^)-dependent lysine deacylases, with Sirtuin 5 (SIRT5) and Sirtuin 7 (SIRT7) demonstrating significant desuccinylase activity [32].

SIRT5 is the first identified desuccinylase and is primarily localized in mitochondria [33,34]. Its structure comprises a Zn^2+^-binding domain and a Rossmann fold domain, which together constitute the binding site for substrates and NAD^+^ [35,36,37]. Unlike other members of the Sirtuin family, SIRT5 possesses two positively charged residues, Tyr102 and Arg105, within its substrate-binding pocket. These residues can form hydrogen bonds and ionic interactions with the carboxyl group of the succinylated lysine substrate [38]. Consequently, SIRT5 exhibits weak deacetylation activity while demonstrating robust desuccinylation activity [39]. Studies have shown that the knockout of the SIRT5 gene results in a significant increase in protein lysine succinylation levels within cells, whereas acetylation levels remain largely unchanged, thereby confirming SIRT5’s specific role in reversing succinylation [40,41].

SIRT7 is predominantly localized in the nucleus, where its desuccinylation activity is particularly pronounced in histone modifications [42]. In cells with silenced SIRT7, the levels of succinylation at various histone sites, including H3K122, are significantly increased. This increase leads to enhanced chromatin compaction and contributes to the DNA damage repair process [43].

#### 2.2.3. Epigenetic Readers

Proteins that recognize succinylation are referred to as “readers.” To date, Glioma-Amplified Sequence-41 (GAS41) is the only identified protein functioning as a succinylation “reader.” The YEATS domain of GAS41 comprises eight antiparallel β-strands and two α-helices, which together form a hydrophobic binding pocket. Within this pocket, a conserved histidine residue acts as the core site for recognizing succinylated lysine (Ksuc). Furthermore, the pocket is capable of tightly enveloping the carbonyl group (C=O) of Ksuc, facilitating subsequent electrostatic interactions. The protonated histidine residue within the YEATS domain establishes a robust electrostatic salt bridge with the negatively charged carboxyl terminus of Ksuc [44], enabling the detection of Ksuc. Upon recognizing and binding to succinylated modifications, GAS41 recruits additional proteins or protein complexes, thereby altering chromatin conformation and ultimately regulating the transcription of target genes.

## 3. Succinylation Impact Metabolic Reprogramming in Tumors

Based on proteomic evidence, succinylation extensively targets key proteins involved in glucose, lipid, and amino acid metabolism, serving as a critical mechanism that drives metabolic reprogramming in cancer cells [31,44,45]. We have summarized the metabolic reprogramming mediated by succinylation in tumors in Table 1.

### 3.1. Succinylation Mediates Metabolic Reprogramming in Tumors

#### 3.1.1. Glucose Metabolism

**Figure 2:**
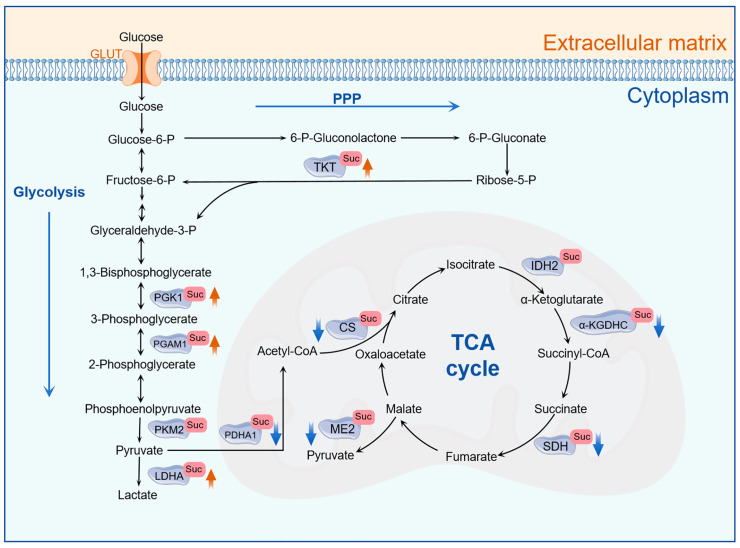
Succinylation play a vital role in metabolic reprogramming. Succinylation in tumor cells plays a crucial role in regulating metabolic enzymes involved in glycolysis, TCA cycle, and the pentose phosphate pathway (PPP). “↑” refers to up-regulated enzyme activity; “↓” refers to down-regulated enzyme activity.

Glucose serves as a primary energy source for tumor cells. Extracellular glucose is transported into cells via glucose transporters (GLUTs) [69]. Within tumor cells, glucose is metabolized through complete oxidative pathways, primarily involving glycolysis, TCA cycle, and the pentose phosphate pathway (PPP), thereby providing essential energy for cancer cells [56,70,71]. Increasing evidence underscores the significance of succinylation of key enzymes in metabolic pathways, which plays a crucial role in reshaping tumor cell metabolism (see Figure 2 and Table 1).

Succinylation exerts a dual regulatory effect on enzyme activity; specifically, the succinylation of certain enzymes enhances their catalytic activity. In lung cancer, P300-mediated succinylation of phosphoglycerate kinase 1 (PGK1) enhances PGK1 enzymatic activity, consequently promoting glycolysis and driving the malignant progression of lung cancer [46]. In hepatocellular carcinoma (HCC), HAT1-catalyzed succinylation at K99 of phosphoglycerate mutase 1 (PGAM1) significantly augments its enzymatic activity, thereby enhancing glycolysis and facilitating HCC progression [49]. Further studies on related drugs suggest that targeting PGAM1 succinylation may serve as a potential therapeutic strategy for HCC intervention. Aspirin reduces the succinylation level of PGAM1 through the NF-κB/HAT1/PGAM1 signaling axis, thereby inhibiting PGAM1 enzymatic activity and glycolysis [50]. Meanwhile, Astragaloside IV restricts PGAM1 enzymatic activity and downregulates glycolysis by inhibiting KAT2A-mediated succinylation of PGAM1 at K161, ultimately suppressing HCC growth [51]. In lung cancer, SIRT5-mediated desuccinylation of pyruvate kinase M2 (PKM2) inhibits its enzymatic activity, redirecting glucose flux into the PPP to enhance antioxidant capacity and assist cells in resisting oxidative stress [54]. Transketolase (TKT) is a crucial enzyme in the non-oxidative phase of the PPP and serves as a central metabolic node linking the PPP with glycolysis [72]. In breast cancer (BC) cells, the succinylation of TKT enhances its enzymatic activity, facilitates the conversion of PPP intermediates into the glycolytic pathway, and ultimately drives the metastasis and invasion of BC cells [55]. Lactate dehydrogenase A (LDHA) is highly expressed in various malignant tumors [73,74]. Succinylation of LDHA at K118 significantly enhances LDHA enzyme activity, accelerates lactate accumulation, and increases the migration and invasion of prostate cancer (PCa) cells. Furthermore, SIRT5 mediates the desuccinylation of LDHA-K118, which could serve as a novel target to prevent PCa progression [58].

Conversely, for certain enzymes, succinylation can inhibit enzymatic activity, whereas the removal of succinyl groups facilitates enzyme activation. In gastric cancer (GC), the succinylation of PKM2 at K475 residue, catalyzed by KAT2A, inhibits PKM2 activity. This inhibition leads to the accumulation of glycolytic intermediates and their diversion into biosynthetic pathways, thereby supporting the rapid proliferation of GC cells [53]. The succinylation of citrate synthase (CS) at K393 and K395 significantly suppresses its enzymatic activity, consequently impeding the proliferation and migration of colon cancer cells [59]. Numerous studies have identified SIRT5 as a critical regulator of tumor metabolic reprogramming. The downregulation of SIRT5 expression results in increased succinylation of α-KGDHC, which reduces enzyme activity, decreases ATP synthesis, and increases the accumulation of reactive oxygen species (ROS), ultimately inhibiting the proliferation and migration of GC cells [60]. Furthermore, the downregulation of SIRT5 enhances the succinylation of pyruvate dehydrogenase A1 (PDHA1), inhibiting its activity and preventing pyruvate from entering the TCA cycle. Instead, pyruvate accumulates and is converted into lactate, further strengthening the Warburg effect in clear cell renal cell carcinoma (ccRCC) [56]. SIRT5-mediated desuccinylation of succinate dehydrogenase complex flavoprotein subunit A (SDHA) enhances its activity, optimizing the efficiency of the TCA cycle and the electron transport chain, thereby promoting tumor energy metabolism and proliferation [61]. Additionally, SIRT5-mediated desuccinylation of mitochondrial malic enzyme 2 (ME2) activates its enzymatic activity, enhances mitochondrial respiration, and aids cells in coping with glutamine deficiency, thereby supporting the development of colorectal cancer (CRC) [6].

In summary, the regulation of metabolic enzyme activity through succinylation demonstrates a nuanced and bidirectional nature, with the potential to either activate or inhibit enzymatic activity. This dynamic modulation ultimately results in the reconfiguration of glucose metabolism in tumor cells. Furthermore, SIRT5-mediated desuccinylation acts as a pivotal negative regulatory switch, contributing to a dynamic and reversible regulatory network.

Succinylation can mediate glucose metabolic reprogramming in tumor cells by influencing protein stability. In triple-negative breast cancer (TNBC) and glioblastoma (GBM), the succinylation of PGK1 competitively inhibits its ubiquitination and subsequent proteasomal degradation, thereby enhancing PGK1 stability, increasing glycolytic flux, and providing sufficient energy for rapid tumor proliferation [47,48]. In GC, the succinylation of LDHA weakens its binding capacity to the selective autophagy adaptor protein 1 (SQSTM1), protecting LDHA from lysosomal degradation, enhancing protein stability, and promoting GC cell proliferation and invasion [57]. In colon cancer, the succinylation of PKM2 at K433 promotes its translocation to the mitochondria, where it binds to voltage-dependent anion channel 3 (VDAC3), inhibiting VDAC3 ubiquitination and degradation, enhancing mitochondrial permeability and ATP production, and sustaining cell survival [52]. Additionally, the serine beta-lactamase-like protein (LACTB) is a protease localized in the mitochondrial intermembrane space that can suppress mitochondrial function by degrading mitochondrial enzymes such as phosphatidylserine decarboxylase (PISD), lysophosphatidylethanolamines (LPEs), and phosphatidylethanolamines (PEs) [75]. OXCT1 mediates the succinylation of LACTB K284, which inhibits its proteolytic activity, thereby enhancing mitochondrial respiration and promoting the progression of HCC [5]. In summary, succinylation regulates protein stability through multiple mechanisms, serving as a critical strategy for tumor cells to sustain elevated levels of glycolytic metabolism.

#### 3.1.2. Lipid Metabolism

Tumor lipid metabolic reprogramming is characterized by aberrantly elevated lipid synthesis [76]. SREBP1a serves as a crucial transcription factor in the biogenesis of cholesterol, fatty acids, and triglycerides [77]. The transcriptional activity of SREBP1a is positively regulated through methylation mediated by protein arginine methyltransferase 5 (PRMT5). The desuccinylation of PRMT5 at K387, catalyzed by SIRT7, enhances its methyltransferase activity, thereby increasing the transcriptional function of SREBP1a. This process ultimately promotes lipid metabolic reprogramming and drives tumor progression [62]. Fatty acid β-oxidation is a critical catabolic pathway essential for maintaining cellular energy homeostasis, occurring in both mitochondria and peroxisomes [78]. Acyl-CoA oxidase 1 (ACOX1) acts as the rate-limiting enzyme in peroxisomal fatty acid β-oxidation [79]. SIRT5-mediated desuccinylation of ACOX1 inhibits the formation of its active dimer, consequently reducing its enzymatic activity. Knocking down SIRT5 results in a 1.5-fold increase in ACOX1 activity and enhances the fatty acid catabolic flux in HCC [63]. Consequently, enzymes involved in tumor lipid synthesis and metabolism are regulated by lysine succinylation.

Tumor cells can utilize ketone bodies as an alternative energy source to cope with energy stress. The synthesis and catabolism of ketone bodies are precisely regulated by succinylation modifications. A key step in ketone body synthesis is catalyzed by human mitochondrial hydroxymethylglutaryl-CoA synthase 2 (HMGCS2) [80]. The enzymatic activity of HMGCS2 is activated through SIRT5-mediated desuccinylation, promoting ketone body production [64]. During ketolysis, OXCT1 converts acetoacetate and succinyl-CoA into acetoacetyl-CoA and succinate, thereby linking ketone body metabolism with the TCA cycle. Recent studies have revealed that OXCT1 interacts with SUCLA2, which catalyzes the synthesis of succinyl-CoA. The locally accumulated succinyl-CoA not only serves as a substrate to promote ketolysis but also induces the succinylation of OXCT1 at K421. This modification significantly enhances the enzymatic activity of OXCT1, accelerates ketolysis, promotes the generation of acetyl-CoA and ATP, and facilitates HCC cell growth. Inhibition of OXCT1 succinylation effectively slows tumor growth [65]. In conclusion, both the synthesis and catabolism of ketone bodies are finely regulated by succinylation modifications.

#### 3.1.3. Amino Acid Metabolism

Certain tumors exhibit a pronounced dependence on glutamine rather than glucose during proliferation, a phenomenon known as “glutamine addiction” [81,82]. The catabolism of glutamine begins with a deamination reaction catalyzed by glutaminase (GLS). The resulting glutamate can be further converted into α-KG, which subsequently enters the TCA cycle to facilitate ATP production [83]. The activity of GLS is dynamically regulated through succinylation. In PDAC, the phosphorylation of SUCLA2 results in its dissociation from GLS, leading to the succinylation of GLS at K311. This modification induces GLS oligomerization, significantly enhancing its enzymatic activity. Consequently, this accelerates glutamine metabolism and promotes the production of antioxidant substances such as NADPH and glutathione, thereby enhancing the resistance of tumor cells to oxidative stress [66]. In BC, SIRT5-mediated desuccinylation of GLS at K164 residue hinders the ubiquitination of GLS at K158 and subsequent proteasomal degradation, thereby increasing GLS stability and ultimately supporting tumor cell proliferation [67].

Additionally, serine catabolism is regulated by succinylation. The metabolism of serine is catalyzed by serine hydroxymethyltransferase 2 (SHMT2) [84]. In CRC and osteosarcoma (OS) cells, SIRT5 facilitates the desuccinylation of SHMT2 at K280, thereby enhancing its activity and promoting serine catabolism, which subsequently drives tumor progression [68].

### 3.2. Succinylation-Mediated Metabolic Reprogramming Reshapes Tumor Immunity

The malignant growth of cancer cells is intricately linked to tumor immune evasion, with alterations in the metabolic state of tumor cells acting as a critical driver of this phenomenon [85]. Recent studies have demonstrated that OXCT1 mediates the succinylation of PGK1 at K146, which competitively inhibits its ubiquitination-mediated degradation, thereby enhancing PGK1 protein stability. The stabilized PGK1 subsequently upregulates the expression of programmed death-ligand 1 (PD-L1), thereby reinforcing tumor cell resistance to T cell-mediated cytotoxicity. Consequently, OXCT1-mediated succinylation of PGK1 not only promotes cell proliferation by modulating glycolytic flux but also drives immune evasion in triple-negative breast cancer through the enhancement of PGK1 stability [47].

Furthermore, aerobic glycolysis results in hypoxia and an acidic tumor microenvironment. Under hypoxic conditions, this can lead to the reversal of succinate dehydrogenase (SDH) activity and the inhibition of respiratory chain function, causing the accumulation of succinate within mitochondria [86]. Succinate competitively inhibits prolyl hydroxylases, thereby stabilizing hypoxia-inducible factor-1α (HIF-1α) protein. The stabilized HIF-1α subsequently transcriptionally activates multiple pro-inflammatory cytokines, including interleukin-1β (IL-1β) and interferon-γ (IFN-γ) [87]. Sustained high levels of inflammatory factors such as IL-1β induce macrophage polarization toward the pro-inflammatory M1 phenotype, reshaping the immunosuppressive microenvironment and ultimately promoting tumor growth and malignant progression [88].

In summary, succinylation reshapes tumor immunity through a dual mechanism. On one hand, it promotes tumor immune escape by regulating the stability of key metabolic enzymes and upregulating the expression of immune checkpoints. On the other hand, the accumulation of metabolites, such as succinate, mediated by succinylation activates sustained inflammatory signaling and induces functional reprogramming of immune cells, thereby systematically shaping an immunosuppressive microenvironment. The metabolic reprogramming mediated by succinylation profoundly alters the landscape of tumor immunity.

## 4. Succinylation Affect Epigenetic Remodeling in Tumors

**Figure 3:**
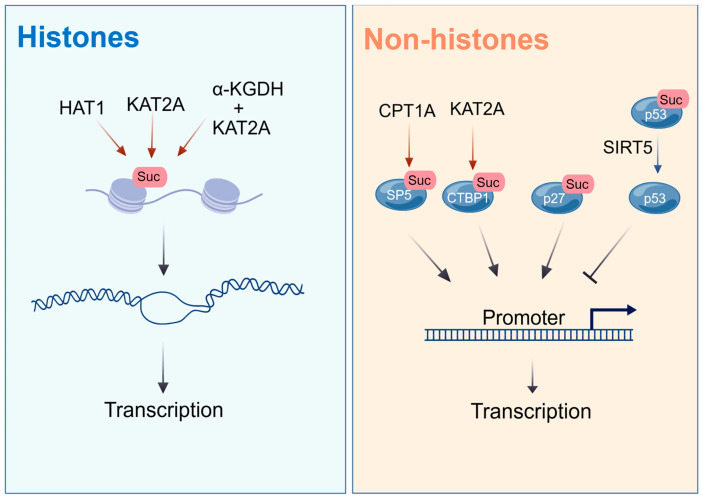
The role of succinylation in epigenetic remodeling. Histone succinylation modulates chromatin structure to enhance gene expression and DNA repair mechanisms in diverse tumors. Additionally, non-histone succinylation impacts cancer advancement by modulating the activity of transcription factors and subsequent gene expression.

Succinylation plays a critical role in epigenetic remodeling by regulating the functions of both histones and non-histone proteins [11,89], which modulates gene expression and influences cancer progression [8,90] (see Figure 3).

### 4.1. Succinylation of Histones

Histone succinylation regulates chromatin structure and gene transcription by altering the charge of histone lysine residues, which weakens the electrostatic interactions between DNA and histones [91,92]. This form of epigenetic regulation, mediated by succinylation, is particularly significant in transcription initiation regions [91,93]. For instance, the succinylation of Histone H3 at K122 by p300/CBP reduces nucleosome stability, enhances DNA accessibility, and increases transcriptional activity [27]. Conversely, SIRT5 (or SIRT7) regulates desuccinylation at H3K122, leading to chromatin stabilization and compaction, thereby suppressing transcription [27,43].

Histone succinylation plays a critical role in various tumors by regulating chromatin states that influence gene expression and DNA repair. In HCC, HAT1-mediated succinylation of H3K122 promotes the expression of genes such as CREBBP, thereby driving cell proliferation [49]. In hepatitis B virus (HBV)-infected cells, SIRT7-mediated desuccinylation of H3K122, which is associated with viral cccDNA, suppresses HBV transcription and replication, consequently reducing the risk of HCC development [32]. In PDAC, KAT2A catalyzes succinylation at H3K79 in the promoter region of YWHAZ, enhancing the transcription and expression of YWHAZ (which encodes 14-3-3ζ), subsequently promoting glycolysis and proliferation by stabilizing β-catenin [28]. In glioblastoma, α-KGDH binds to KAT2A, inducing succinylation of H3K79 and activating the transcription of pro-oncogenic genes, including PI3K and c-Jun [23]. During the DNA damage response, the succinylation of H2A.X locally opens chromatin, facilitating the recruitment of DNA repair factors [94].

### 4.2. Succinylation of Non-Histone

Succinylation serves as a critical mechanism influencing cancer progression by regulating transcription factor activity and downstream gene expression. In PCa, CPT1A mediates the succinylation of specificity protein 5 (SP5) at K391, significantly enhancing SP5’s binding capacity to the promoter of 3-phosphoinositide-dependent protein kinase 1 (PDPK1), thereby promoting tumor cell glycolysis through the activation of the AKT/mTOR signaling pathway [95]. Additionally, KAT2A catalyzes the succinylation of the transcriptional repressor CTBP1 at K46 and K280. This modification attenuates CTBP1’s transcriptional repression of the CDH1 gene, consequently driving prostate cancer metastasis [96]. In colorectal cancer, SIRT5 mediates the desuccinylation of p53 at K120, inhibiting its transcriptional activity and the expression of downstream genes such as p21 [97]. In lung cancer, p23 undergoes non-enzymatic succinylation at K7 and K33, which drives its nuclear translocation, enhancing COX-2 transcription and ultimately promoting tumor growth [98]. These studies collectively reveal the central role of succinylation in the regulatory network of transcription factors.

## 5. Crosstalk Between Succinylation and Other PTMs

Many identified succinylation sites overlap with other PTMs sites, suggesting potential cross-interactions between succinylation and other PTMs, including acetylation, methylation, ubiquitination, and glycosylation [99,100]. These PTMs are also crucial in the development and progression of cancer.

### 5.1. Acetylation

Protein acetylation and succinylation exhibit significant overlap in their regulatory factors and substrate targets [18,101]. Treatment of HCT116 cells with dichloroacetate (DCA) revealed that multiple enzymes in the tricarboxylic acid (TCA) cycle are coordinately regulated by acetylation and succinylation, which modulates their enzymatic activity and mediates the anticancer mechanism of DCA [102]. Nucleophosmin (NPM1) undergoes both acetylation and succinylation at lysine residues, influencing the DNA damage response (DDR) in breast cancer through the regulation of chromatin structure [94].

### 5.2. Methylation

Protein methylation may establish a complex regulatory network with succinylation during tumor growth and metastasis. For instance, H3K9me2 serves as a common marker for transcriptional repression in the progression of malignant tumors [103]. In esophageal squamous cell carcinoma (ESCC) cells, succinylation may negatively regulate H3K9me2, thereby attenuating H3K9me2-mediated transcriptional repression and promoting the invasion and metastasis of ESCC [99].

### 5.3. Ubiquitination

Protein homeostasis is meticulously regulated by post-translational modification networks, in which the antagonistic relationship between ubiquitination and succinylation regarding protein stability constitutes a critical mechanism. For instance, succinylation of S100A10 at K47 inhibits its ubiquitination by competing for the same lysine residue, thereby reducing protein degradation and enhancing stability [104]. Furthermore, the succinylation occupancy of GLS at K158 and K164 prevents its ubiquitination at K48, consequently inhibiting subsequent proteasomal degradation [105].

### 5.4. GlcNAcylation

GlcNAcylation refers to the process of attaching N-acetylglucosamine (GlcNAc) to serine or threonine residues of substrate proteins through O-glycosidic bonds. Elevated levels of O-GlcNAc have been observed in various cancers, where they play a crucial role in regulating tumor metabolism, growth, and metastasis [106,107,108]. Notably, O-GlcNAcylation and succinylation exert opposing regulatory effects on immune checkpoints in cancer cells. Specifically, succinylation of PD-L1 at K129 induces its degradation via the endosome-lysosome pathway, thereby enhancing anti-tumor immune responses [109]. Conversely, O-GlcNAcylation inhibits the lysosomal degradation of PD-L1, leading to increased PD-L1 expression and promoting tumor immune escape [110]. The mutual antagonism between these two modifications in regulating PD-L1 stability exemplifies the intricate balance of post-translational modification networks during tumor progression.

## 6. Targeted Drug

Several inhibitors targeting succinylation modifications have been identified to exhibit anti-tumor potential. For instance, HSP90 inhibitors, such as AUY922, may exert anti-tumor effects by influencing the succinylation of H4K20 and H3K122 in bladder cancer [111]. HSP90 inhibitors bind to HSP90, inhibiting its function as a molecular chaperone that assists client proteins in proper folding and stability maintenance. This leads to the degradation or altered activity of numerous acylation regulatory factors. It is important to note that HSP90 inhibitors are not specifically designed to target succinylation modification. Compound 8 inhibits the succinylation of PKM2 at the K433 site, blocks PKM2 mitochondrial translocation, and disrupts the interaction between PKM2 and voltage-dependent anion channel 3 (VDAC3). This results in VDAC3 degradation, decreased mitochondrial permeability, reduced ATP production, and ultimately induces tumor cell death [52]. Compound 8 acts as an inhibitor of PKM2 protein activity. By suppressing the overall function of PKM2, Compound 8 blocks its subsequent succinylation-dependent mitochondrial translocation, though it does not directly interfere with the succinylation modification process itself [112]. SIRT5 inhibitors (DK1-04e and MC3482) impede the initiation and progression of breast cancer by inhibiting the desuccinylation of various metabolic enzymes, including IDH and GLS [41,113]. The effect of these inhibitors results in a global elevation of protein succinylation levels within cells. Currently, anticancer drugs targeting lysine succinylation remain in the preclinical research stage, but their potential to improve patient outcomes has been preliminarily demonstrated. Therefore, designing inhibitors capable of precisely modulating this modification remains a central objective in the field of drug discovery.

In addition to new drug development, certain clinical drugs may exert antitumor effects by modulating protein succinylation levels. For instance, aspirin-induced desuccinylation of PGAM1 at K99 significantly reduces the enzymatic activity of PGAM1, thereby directly blocking the glycolytic pathway. The impaired glycolysis subsequently leads to a global decrease in protein succinylation levels in HCC cells (HepG2, Huh7), ultimately inhibiting tumor cell glycolysis and proliferation [50]. The lipid-lowering drug Bezafibrate upregulates the succinyltransferase CPT1A by activating the PPAR/PGC1α pathway. CPT1A catalyzes the succinylation of PD-L1 at K129, resulting in the degradation of PD-L1 via the endosome-lysosome pathway. This process alleviates PD-L1-mediated suppression of CD8+ T cells and enhances T cell-mediated tumor killing [99]. Dichloroacetate (DCA), a pyruvate dehydrogenase kinase (PDK) inhibitor, enhances mitochondrial glucose oxidation, leading to an expanded succinyl-CoA pool. This triggers extensive succinylation modifications of mitochondrial metabolic enzymes, remodels oxidative phosphorylation function, and suppresses the growth of colon cancer cells [102]. Lovastatin can inhibit lung metastasis of triple-negative breast cancer stem cells (CSCs) by regulating the succinylation of cytoskeletal proteins [114]. Additionally, the traditional Chinese medicine component catalpol can significantly influence various modification levels in breast cancer, including succinylation [115]. This collective evidence suggests that targeting succinylation modification is one of the potential mechanisms through which existing drugs exert their antitumor effects and provides a basis for developing novel therapeutic strategies based on succinylation regulation. However, these drugs do not specifically target succinylation modification, and their mechanisms of action may involve multi-pathway regulation. Furthermore, the current evidence primarily derives from cell and animal studies and has not yet progressed to human clinical trials. Thus, the efficacy and safety of these approaches still require further validation.

## 7. Conclusions

Succinylation, a critical PTMs of proteins, has been demonstrated to play a significant role in tumor initiation and progression. This article systematically reviews the history of its discovery, key regulatory enzymes, and essential mechanistic roles in tumors. By dynamically modulating chromatin structure, gene transcription, metabolic enzyme activity, and protein stability, succinylation profoundly influences tumor metabolic reprogramming and epigenetic remodeling, serving as a pivotal link between cellular metabolism and epigenetic regulation.

Notably, the regulatory effect of succinylation on metabolic enzyme activity is context-dependent. In the TCA cycle, succinylation generally inhibits the activity of related enzymes, while desuccinylation activates them. This contrasts with the regulatory patterns observed in glycolysis and the PPP. Furthermore, the same protein may exert opposite functions in different tumors. For instance, the activity of PKM2 is inhibited by succinylation in gastric cancer but activated in lung cancer, indicating that succinylation-mediated regulation of enzyme activity is contingent upon tumor type and microenvironment [53,54].

Current studies highlight extensive crosstalk between succinylation and other modifications; however, the complete regulatory network and underlying mechanisms remain to be fully elucidated. Additionally, beyond histone acylation modifications, epigenetic regulation also includes DNA methylation, RNA modifications, and non-coding RNAs (ncRNAs) [116]. The interplay between succinylation and these modifications further complicates the epigenetic regulatory landscape. To deeply elucidate the dynamic interplay between succinylation and other post-translational modifications, it is essential to develop novel targeting strategies based on succinylation regulation, explore succinylation modification biomarkers, and provide innovative approaches for precise tumor intervention and therapeutic efficacy monitoring.

## Figures and Tables

**Table 1:** Succinylation impact metabolic reprogramming in tumor cells.

Protein	Ksucc Sited	Regulatory Enzymes	Tumor	Mechanism	References
PGK1	/	p300	Lung cancer	Promotes glucose metastasis	[46]
	K146	OXCT1	Triple negative breast cancer	promotes aerobic glycolysis and immune escape	[47]
	K191/K192	/	Glioblastoma	Promotes cell growth and aerobic glycolysis	[48]
PGAM1	K99	HAT1	Hepatocellular carcinoma	Promotes the PGAM1 activities and glycolysis	[49,50]
	K161	KAT2A	Hepatocellular carcinoma	Astragaloside IV inhibits cell viability and glycolysis	[51]
PKM2	K433	/	Colon cancer	Promotes mitochondrial translocation of PKM2	[52]
	K475	KAT2A	Gastric cancer	Promotes cell growth and glycolysis	[53]
	K498	SIRT5	Lung cancer	Promotes antioxidant response and tumor growth	[54]
TKT	/	/	Breast cancer	Promotes the metastasis and invasion of BC	[55]
PDHA1	K351	SIRT5	Clear cell renal cell carcinoma	Downregulation of SIRT5 enhances the Warburg effec	[56]
LDHA	K222	CPT1A	Gastric cancer	Enhances protein stability and cell proliferation	[57]
	K118	SIRT5	Prostate cancer	Decrease in the level of SIRT5 promotes PCa progression	[58]
CS	K393/K395	SIRT5	Colon cancer	Promotes cell proliferation and migration	[59]
IDH2	/	SIRT5	Breast cancer	Knockdown of SIRT5 inhibits tumor progressio	[41]
α-KGDHC	/	SIRT5	Gastric cancer	Suppresses GC cell growth and migration	[60]
SDH	K547	SIRT5	Clear cell renal cell carcinoma	Promotes metabolism and proliferation	[61]
ME2	K346	SIRT5	Colorectal cancer	Promotes CRC progression	[6]
LACTB	K284	OXCT1	Hepatocellular carcinoma	Enhance mitochondrial respiration and promote HCC progression	[5]
PRMT5	K387	SIRT7	Hepatocellular carcinoma	Promotes lipid metabolism and tumor growth	[62]
ACOX1	/	SIRT5	Hepatocellular carcinoma	SIRT5 downregulation increases ACOX1 activity and oxidative DNA damage response	[63]
HMGCS2	K83/K310	SIRT5	Hepatocellular carcinoma	Promotes fatty acid β-oxidation and ketone body synthesis	[64]
OXCT1	K421	/	Hepatocellular carcinoma	Enhances ketolysis and tumor growth	[65]
GLS	K311	/	Pancreatic ductal adenocarcinoma	Promotes tumor cell survival and tumor growth	[66]
	K164	SIRT5	Breast cancer	Promotes metabolism and proliferation	[67]
SHMT2	K280	SIRT5	Colorectal cancer and Osteosarcoma	Promotes metabolism and proliferation	[68]

“/” refers to no data. Abbreviations: ACOX1, acyl-CoA oxidase 1; α-KGDHC, α-ketoglutarate dehydrogenase complex; CS, citrate synthase; CPT1A, carnitine palmitoyltransferase 1A; GLS, glutaminase; HAT1, histone acetyltransferase 1; HMGCS2, protein arginine methyltransferase 5; IDH2, Isocitrate Dehydrogenase (NADP(+)) 2; KAT2A, lysine acetyltransferase 2A; LACTB, beta-lactamase-like protein; LDHA, lactate dehydrogenase A; ME2, Malic enzyme 2; OXCT1, 3-oxoacid CoA-transferase 1; PGK1, phosphoglycerate kinase 1; PGAM1, phosphoglycerate mutase 1; p300, E1A binding protein P300; PKM2, pyruvate kinase M2; PDHA1, pyruvate dehydrogenase E1 subunit alpha 1; PRMT5, protein arginine methyltransferase 5; SIRT5, sirtuin 5; SIRT7, sirtuin 7; SDH, succinate dehydrogenase; SHMT2, serine hydroxymethyltransferase-2; TKT, transketolase.

## Data Availability

No new data were created or analyzed in this study. Data sharing is not applicable to this article.

## Abbreviations

The following abbreviations are used in this manuscript:

PTM	Post-translational modification
Ksuc	Lysine succinylation
TCA cycle	Tricarboxylic acid cycle
α-KG	α-ketoglutarate
SDH	Succinate dehydrogenase
α-KGDHC	α-ketoglutarate dehydrogenase complex
Val	Valine
Ile	Isoleucine
Met	Methionine
Thy	Thymine
OCFAs	β-oxidation of odd-chain fatty acids
SUCLA2	succinyl-CoA ligase ADP-forming subunit β
HAT1	Histone acetyltransferase 1
KAT2A	Lysine acetyltransferase 2A
PDAC	Pancreatic ductal adenocarcinoma
CPT1A	Carnitine palmitoyltransferase 1A
OXCT1	3-oxoacid CoA-transferase 1
LACTB	Serine beta-lactamase-like protein
SIRT5	Sirtuin 5
SIRT7	Sirtuin 7
GLUTs	Glucose transporters
PPP	Pentose phosphate pathway
PGK1	Phosphoglycerate kinase 1
PGAM1	Phosphoglycerate mutase 1
HCC	Hepatocellular carcinoma
PKM2	Pyruvate kinase M2
TKT	Transketolase
BC	Breast cancer
LDHA	Lactate dehydrogenase A
PCa	Prostate cancer
GC	Gastric cancer
CS	Citrate synthase
ROS	Reactive oxygen species
PDHA1	Pyruvate dehydrogenase A1
ccRCC	Clear cell renal cell carcinoma
SDHA	Succinate dehydrogenase complex flavoprotein subunit A
ME2	Malic enzyme 2
CRC	Colorectal cancer
TNBC	Triple-negative breast cance
GBM	Glioblastoma
SQSTM1	Selective autophagy adaptor protein 1
VDAC3	Voltage-dependent anion channel 3
PRMT5	Protein arginine methyltransferase
ACOX1	Acyl-CoA oxidase 1
HMGCS2	Hydroxymethylglutaryl-CoA synthase 2
GLS	Glutaminase
SHMT2	Serine hydroxymethyltransferase 2
OS	Osteosarcoma
HBV	Hepatitis B virus
SP5	Specificity protein 5
PDPK1	3-phosphoinositide-dependent protein kinase 1
DCA	Dichloroacetate
NPM1	Nucleophosmin
DDR	DNA damage response
ESCC	Esophageal squamous cell carcinoma
GlcNAc	N-acetylglucosamine
CSCs	Triple-negative breast cancer stem cells
ncRNAs	non-coding RNAs
